# Aging on a different scale – chronological versus pathology-related aging

**DOI:** 10.18632/aging.100606

**Published:** 2013-10-16

**Authors:** Joost P.M. Melis, Martijs J. Jonker, Jan Vijg, Jan H.J. Hoeijmakers, Timo M. Breit, Harry van Steeg

**Affiliations:** ^1^ National Institute for Public Health and the Environment (RIVM), Center for Health Protection, Bilthoven, the Netherlands; ^2^ Leiden University Medical Center, Department of Toxicogenetics, Leiden, the Netherlands; ^3^ University of Amsterdam (UvA), MicroArray Department & Integrative Bioinformatics Unit (MAD-IBU), Swammerdam Institute for Life Sciences (SILS), Faculty of Science (FNWI), Amsterdam, the Netherlands; ^4^ Netherlands Bioinformatics Centre (NBIC), Nijmegen, the Netherlands; ^5^ Albert Einstein College of Medicine, Department of Genetics, New York, USA; ^6^ Erasmus University Medical Center, CGC Department of Genetics, Rotterdam, the Netherlands

**Keywords:** aging, senescence, diseases, signaling pathways, metabolism, lipofuscin

## Abstract

In the next decades the elderly population will increase dramatically, demanding appropriate solutions in health care and aging research focusing on healthy aging to prevent high burdens and costs in health care. For this, research targeting tissue-specific and individual aging is paramount to make the necessary progression in aging research. In a recently published study we have attempted to make a step interpreting aging data on chronological as well as pathological scale. For this, we sampled five major tissues at regular time intervals during the entire C57BL/6J murine lifespan from a controlled in vivo aging study, measured the whole transcriptome and incorporated temporal as well as physical health aspects into the analyses. In total, we used 18 different age-related pathological parameters and transcriptomic profiles of liver, kidney, spleen, lung and brain and created a database that can now be used for a broad systems biology approach. In our study, we focused on the dynamics of biological processes during chronological aging and the comparison between chronological and pathology-related aging.

## INTRODUCTION

Aging is generally considered the result of time-dependent deterioration due to stochastic, accumulative ‘wear and tear’ causing gradual degeneration. Therefore, time is the prevailing determinant in age-related processes [1, 2]. However, although aging is highly correlated with time, additional factors significantly influence the rate of aging and as a consequence, individual aging differs greatly [3-6]. Moreover, the rate of age-related deterioration and functional decline varies within every individual in a tissue-specific manner [7-12]. In humans, lifespan ranges from less than 10 years for the severe progeria patients [13, 14] to over 100 years for centenarians. Many pro-aging factors are likely controlled to some extent by genetic variation [2, 15]. However, even in genetically identical, inbred animals aging rate varies substantially among individuals [4, 16, 17]. This indicates that other factors besides time are of significance. Genomic instability due to accumulation of stochastic damage in DNA over time [16-28] causing cell death and cellular senescence is believed to be one of the drivers of aging [29-43]. It has proved difficult to mechanistically dissect processes involved in individual and tissue-specific aging.

### Dynamics in aging, chronological aging and pathology-related aging

To investigate general health deterioration and loss of homeostasis in aging, we attempted to determine 1) the dynamics of biological processes during aging and 2) correlate patho-physiological aging end points to transcriptomic responses, which are generally believed to determine the cellular phenotype [44]. Previously, large scale studies provided valuable new insights into aging mechanisms in multiple species, tissues and genotypes [10, 12, 45-52]. Several of these studies focussed on young versus old comparisons [10, 45, 47, 50], making correlation studies difficult to execute.

**Figure 1:**
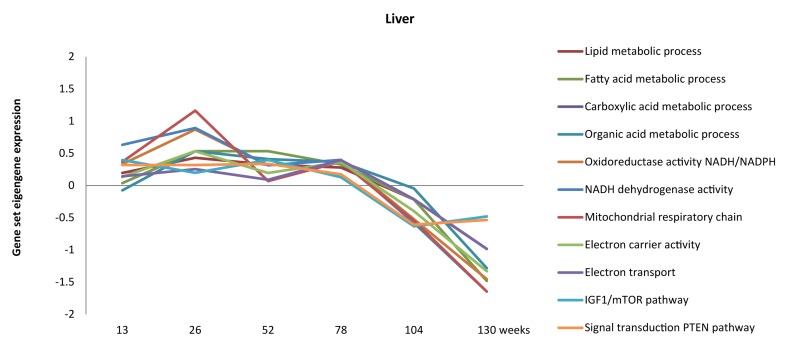
Dynamics of metabolism, energy and mitochondrial-related processes throughout aging in liver (adapted from [4]).

We attempted to fill part of the hiatus between chronological aging rate and its associated patho-physiological patterns in the mouse by full genome gene expression profiling of five organs at six ages covering the entire lifespan in mice [4]. Firstly, using the intercurrent gene expression profiles from the six time points, we were able to follow the dynamics of biological processes during chronological aging. For instance, energy homeostasis, lipid metabolism, IGF-1, PTEN and mitochondrial function in liver were slightly up-regulated during the first half of the lifespan but declined during the last 25% of the lifespan (Figure 1) [4]. These processes have previously been correlated to chronological aging by others [12, 53-67], but interpreting the dynamics of biological functions throughout the lifespan in multiple tissues has been proved difficult so far. Our data can contribute to unravelling the dynamics of functional pathways throughout time in several tissues.

**Figure 2:**
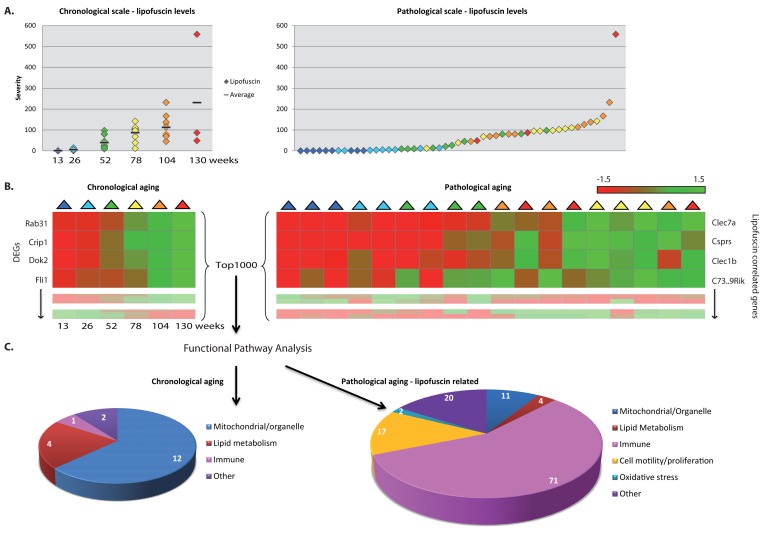
Functional pathways analyses on chronological and pathology-related scale. (**A**) Pathological age-related parameter lipofuscin accumulation was scored at regular intervals during aging. On average per age group lipofuscin levels increase with aging (left panel), however individual differences between chronological ages are notable (right panel). (**B**) Gene expression profiles were investigated according to a chronological scale (left panel, DEGs = differentially expressed genes) and pathological scale (right panel). For the latter, gene expression profiles are ranked according to correlation to the severity of the pathological parameter. (**C**) Functional genomics analyses using the top1000 of either chronological age-related genes or pathology-related genes indicate that besides overlapping responses, also differences are visible, *e.g.* immune response appeared highly correlated to hepatic lipofuscin accumulation. Numbers in diagrams represent the number of pathway hits.

Besides focusing on the dynamics of processes during chronological aging, we additionally scored a range of age-related pathologies (n=18, of which some are possibly novel as an aging-marker, Table 1) in a systematic fashion over time in these five organs to address pathology-related aging. The age-related pathological parameters shown in Table 1 changed over time per age group; nevertheless, substantial individual variation was found (exemplified for instance by the hepatic lipofuscin accumulation in Figure 2A). Not only age-related pathological findings were tissue-specific, but the overall age-related changes in the gene expression profiles were also highly tissue-specific, arguing for caution to consider aging as a systemic generic process and take into account tissue-specific aging [7-12, 68-73].

To relate biological functions to age-related patho-physiological end point, we substituted chronological time for the severity of the scored age-related pathological variables in each tissue, as is demonstrated in Figure 2A for lipofuscin accumulation in liver. On a pathological scale, a liver sample of a 2 year old mouse (orange) could be considered younger than the liver of a 1 year old mouse (green) for a certain pathological parameter when the severity of this pathological condition was lower in the 2 year old sample.

**Figure 3:**
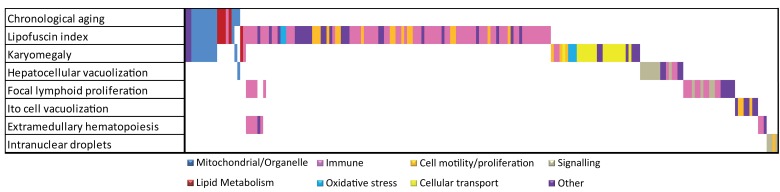
Overlap analysis of functional responses in chronological and pathology-related aging. Summarized Metacore GeneGO pathways and GO responses are color coded. For chronological and each pathological parameter in liver the functional responses are plotted. Overlapping bars represent overlapping functional responses, e.g. the majority of mitochondrial/organelle-related responses are related to chronological aging, lipofuscin accumulation and karyomegaly. Immune responses are correlated to several age-related pathologies in liver.

Pathology-related transcriptomic profiles were generated by ranking gene expression profiles based on their correlation with the degree of age-related pathologies (Figure 2B, right panel) and subsequently assessing the biological functions of those correlated gene expression profiles (Figure 2C, right panel). These analyses revealed several biological processes that have previously been found associated with age and appeared in both our chronological and pathology-related aging analyses (Figure 2C, Figure 3). Overlap analysis, based on a ranked top1000 as input between hepatic chronological aging (Fig. 2C, left) and lipofuscin-related output in liver (Fig. 2C, right) yielded overlapping biological pathways functional in mitochondrial processes and lipid metabolism for example. However, also ample differences between aging on a chronological and a pathological scale were apparent. In liver, immune responses, cell motility/proliferation/activation and oxidative stress responses paralleled the kinetics of lipofuscin accumulation, which is a generally accepted biomarker for aging and an indicator of cumulative cellular oxidative stress (Figure 2C) [74-86]. Apparently, the lipofuscin-correlated transcripts were overrepresented in many more functional pathways than the 1000 FDR-ranked chronological genes, resulting in an increase in the number of functional (mostly immune-related) pathways. Immune responses, cell motility/adhesion and oxidative stress have been previously linked to (hepatic) injury and aging [87-98] and according to our results these related processes might be contributing factors to the biological diversity in hepatic injury and aging per individual.

Figure 3 depicts an overlap analysis of functional pathways between chronological and all pathology-related aging parameters for liver (for detailed information on the other tissues see [4]). Results indicate that, besides existing overlap between chronological and pathological aging processes (e.g. mitochondrial processes and lipid metabolism), many divergent functional responses were revealed using a (often tissue-specific) pathological scale. These divergent responses leave us with numerous interesting anchor points for future aging research to correlate age-related biological pathways to actual patho-physiological end-points and reveal possible underlying mechanisms, as exemplified for hepatic lipofuscin accumulation. We hope our results contribute to a new paradigm in aging and medical research taking into account individual and tissue-specific aging levels. For this however, as a next step, a systems biology approach is required to decipher causal age-related mechanisms. Correlating pathophysiological aging endpoints to gene expression and other cellular signatures will become a focus in current aging research to explore loss of homeostasis and general health decline on individual or organ-specific level. We hope that our first step in this direction will inspire other researchers to contribute to resolve these complex processes using an integral multi-disciplinary system biology approach.
